# Antioxidant and phytochemical properties of *Carpobrotus edulis* (L.) bolus leaf used for the management of common infections in HIV/AIDS patients in Eastern Cape Province

**DOI:** 10.1186/1472-6882-12-215

**Published:** 2012-11-09

**Authors:** Beauty E Omoruyi, Graeme Bradley, Anthony J Afolayan

**Affiliations:** 1 Department of Biochemistry and Microbiology, University of Fort Hare, Private Bag X1314, Alice 5700, South Africa; 2 Department of Botany, University of Fort Hare, Private Bag X1314, Alice 5700, South Africa

**Keywords:** Carpobrotus edulis, Solvent extraction, Antioxidant, Free radicals, Phytoconstituents

## Abstract

**Background:**

*Carpobrotus edulis* (Mesembryanthemaceae), also known as igcukuma in Xhosa language is a medicinal plant used by the traditional healers to treat common infections in HIV/AIDS patients. Based on this information, we researched on the plant phytoconstituents, as well as its inhibitory effect using aqueous and three different organic solvent extracts in order to justify its therapeutic usage.

**Methods:**

Antioxidant activity of the extracts were investigated spectrophotometrically against 1,1- diphenyl-2-picrylhydrazyl (DPPH), 2,2’-azino-bis(3-ethylbenzthiazoline-6-sulfonic acid) (ABTS) diammonium salt, hydrogen peroxide (H_2_O_2_), nitric oxide (NO), and ferric reducing power, Total phenols, flavonoids, flavonols, proanthocyanidins, tannins, alkaloids and saponins were also determined using the standard methods.

**Results:**

Quantitative phytochemical analysis of the four solvent extracts revealed a high percentage of phenolics (55.7 ± 0.404%) in the acetone extract, with appreciable amount of proanthocyanidins (86.9 ± 0.005%) and alkaloids (4.5 ± 0.057%) in the aqueous extract, while tannin (48.9 ± 0.28%) and saponin (4.5 ± 0.262%) were major constituents of the ethanol extract. Flavonoids (0.12 ± 0.05%) and flavonols (0.12 ± 0.05%) were found at higher level in the hexane extract in comparison with the other extracts. The leaf extracts demonstrated strong hydrogen peroxide scavenging activity, with the exception of water and ethanol extracts. IC_50_ values of the aqueous and ethanolic extract against DPPH, ABTS, and NO were 0.018 and 0.016; 0.020 and 0.022; 0.05 and 0.023 mg/ml, respectively. The reducing power of the extract was found to be concentration dependent.

**Conclusion:**

The inhibitory effect of the extracts on free radicals may justify the traditional use of this plant in the management of common diseases in HIV/AIDs patients in Eastern Cape Province. Overall, both aqueous and ethanol were found to be the best solvents for antioxidant activity in *C. edulis* leaves.

## Background

Living cells are known to generate free radicals reactive oxygen species (ROS) through physiological and biochemical processes in the body system [1-3]. Free radicals such as OH^-^, O_2_^.-^, ^.^NO^-^, RO_2_^-^ and LOO^-^ are products of normal metabolic processes in the human body. It is true that the body can handle free radicals, but if these radical productions become excessive, it could cause cell wall and DNA damage, leading to chronic diseases like cancers and cardiovascular disease [1-3].

Dietary antioxidant from food intake, such as vitamin E, selenium and polyphenols like green tea has been reported to decrease the adverse effects of free radicals [4]. They act as scavengers by donating one of their own electrons in order to replace the stolen electron from free radicals [4].

Several standard established antioxidant drugs such as butylhydroxytoluene (BHT) and rutin have been reported to be toxic to living cells [5-7]. Rutin drugs also known as quercetin rutinoside is a glycoside of the bioflavonoids used in many countries, including South Africa as medications for the treatment of inflammatory disorders, allergies and viruses [8]. However, specific carcinogenic toxicity has been observed [9]. These include swelling of the throat, tongue, lips or face, chest pain, skin rash etc. BHT drugs, are known to be the most prevalent and approved antioxidant scavengers worldwide, have equally been reported to be toxic to the lungs, even at a lower concentration [5-7].

In recent years, there has been an increasing interest in finding natural antioxidants from medicinal plants [10]. Plants are endowed with free radical scavenging molecules, such as vitamins, terpenoids, phenolic acids, tannins, flavonoids, alkaloids, and other metabolites, which are rich in antioxidant and free radical scavenging properties [11]. In addition, the ingestion of natural antioxidants has shown to enhance the immune defence, reduce risks of cancer, cardiovascular disease, diabetes, and other diseases associated with ageing [12,13].

*Carpobrotus edulis* (L.) Bolus (Mesembryanthemaceae), also known as igcukuma in Xhosa communities, is an edible easily grown groundcover plant that is widespread in the Eastern Cape of South Africa. It flourishes on sandy soil with thick greenish succulent leaves reaching about 10.8 cm in length. This plant is used by the traditional healers in the above mentioned province to treat tuberculosis, diabetes mellitus, sores, high blood pressure, intestinal worms and constipation. It is possible that this plant may contain some bioactive secondary metabolites that work against opportunistic infections [14].

The antioxidant action of polyphenol compounds depends on their free radical scavenging capacity and its ability to reduce iron [15]. The total polyphenol amounts determined from plant and their corresponding antioxidant activity may vary widely, depending on the extraction solvents applied. For example, aqueous and acetone showed the highest efficiency for extraction of phenols among the various solvents used [16]. Olubunmi and Anthony [16], reported that acetone on its own has the capability of extracting both the polar and non-polar compounds from plant samples. Similarly, Brenes et al. [17] reported that the use of acetone extraction solvent resulted in a complete extraction of phenols from olive oils, when compared with other solvent extracts. Methanol extracts however showed the highest antioxidant activities in seabuckthorn seeds when compared with chloroform and ethyl acetate solvent [18]. Recently, we compared the polyphenol content from various solvents (aqueous, ethanol, acetone and hexane) extract of *C. edulis* leaf. Aqueous and ethanol were found to be the best solvents for antioxidant activity [18].

Limited information exists on antioxidant activity of *Carpobrotus edulis* (L) Bolus. Hanen et al. [19] examined the phytochemical properties of methanolic extracts using different plant parts including the leaf of *C. edulis* but no findings are recorded for the antioxidant activity of the aqueous, ethanol, acetone and hexane extracts considering that successful isolation of bio-compounds from plant material is largely dependent on the type of solvent used in the extraction procedure [20]. Therefore, the aim of this study were (1) to determine the quantitative phytochemical present in various extraction solvents of varying polarities, (2), to determine their antioxidant activities in comparison to the established standard drugs in order to justify its therapeutic usage.

## Methods

### Collection and preparation of the extracts

Fresh leaves of *C. edulis* were collected from the Alice area in the Eastern Cape, SA. The plant was authenticated by Prof. DS Grierson of the Botany Department, University of Fort Hare, where a voucher specimen (Omo 2011/1-Omo 2011/19) was kept.

The leaves were washed with tap water, oven dried at 50°C for 24 h and ground to fine power using a electric blender (Waring Products Division, Torrington, USA). Hundred grams (100.00 g) of finely ground plant material was extracted with 1 L of hexane, acetone, ethanol and distilled water respectively. The containers and contents were vigorously shaken for 48 h (Stuart Scientific Orbital SOI, Essex). Particulate matter was allowed to sediment and the supernatant was filtered using Buchner funnel and Whatman No. 1 filter paper. This process was repeated by re-filtering the supernatant with sterile cotton wool and evaporated using a rotavaporator (R-114; Büchi, New Castle, USA) and decanted into pre-weighed labelled beakers. The different extracts were reconstituted in their various extraction solvents to give the required concentrations needed in this study.

### Preliminary screening of the extract phytochemicals

Initial screening tests of the four extracts were performed to ascertain the presence or absence of phytoconstituents such as phenolic compounds, flavonoids, flavonols, proanthocyanidins, tannins, saponins, and alkaloids using standard procedure described by Alex et al. [21].

### Determination of total phenolic content

Total phenol content in the various extracts was determined by the modified Folin-ciocalteu method of Zovko et al. [22]. An aliquot of 0.5 ml of each extract (1 mg/ml) was mixed with 2.5 ml Folin-Ciocalteu reagent (previously diluted with distilled water 1:10 v/v) and 2 ml (75% w/v) of sodium carbonate (Na_2_CO_3_). The tubes were vortexed for 15 s and allowed to stand for 30 min at 40°C for colour development. Absorbance was then measured at 765 nm using Hewlett Packard, UV/visible light spectrophotometer. Samples of extract were evaluated at a final concentration of 1 mg/ml. Total phenolics content were expressed as mg/g tannic acid equivalent using the following equation from the calibration curve: Y = 0.1216×, R^2^= 0.936512, where × is the absorbance and Y is the tannic acid equivalent in mg/g. The experiment was conducted in triplicate and the results were expressed as mean ± SD values.

### Estimation of total flavonoids

The formations of a complex aluminium chloride colour were estimated by using the method described by Ordonez et al. [23]. Half a ml of various solvent extracts (1 mg/ml) was mixed with 0.5 ml of 2% aluminium chloride (AlCl_3_) prepared in ethanol. The resultant mixture was incubated for 60 min at room temperature for yellow colour development which indicated the presence of flavonoid. The absorbance was measured at 420 nm using UV–VIS spectrophotometer. Extract samples were evaluated at a final concentration of 1 mg/ml. Total flavonoid content was calculated as quercetin equivalent (mg/g) using the following equation based on the calibration curve: Y = 0.255x, R^2^ = 0.9812, where × is the absorbance and Y is the quercetin equivalent.

### Estimation of total flavonols

Total flavonol content was determined by adopting the procedure described by Karunakaran and Kumaran [24]. The reaction mixture consisting of 2 ml of the sample, 2 ml of AlCl_3_ prepared in ethanol and 3 ml of (50 g/l) sodium acetate solution was allowed to incubate for 2.5 h at 20°C. Absorbance at 440 nm was measured. Total flavonol content was calculated as mg/g of quercetin equivalent from the calibration curve using the equation: Y Y = 0.0255×, R^2^ = 0.9812 where × is the absorbance and Y is the quercetin equivalent.

### Determination of total proanthocyanidins content

Determination of proanthocyanidins content was done using the procedure reported by Ashafa et al. [25]. A volume of 0.5 ml of each extract solution was mixed with 3 ml of 4% v/v vanillin prepared in methanol and 1.5 ml of hydrochloric acid and then vortexed. The resulting mixture was allowed to stand for 15 min at room temperature followed by the measurement of the absorbance at 500 nm. Total proanthocyanidin content was expressed as catechin (mg/g) using the following equation of the curve: Y = 0.5825×, R^2^ = 0.9277, where × is the absorbance and Y is the catechin equivalent.

### Tannin determination

Tannin content of the samples was determined according to the modified vanillin-HCl methanol method as described by Noha et al. [26]. The vanillin-HCL reagent was prepared by mixing equal volume of 8% HCl and 1% vanillin in methanol. The reagent was mixed just prior to use. About 0.2 g of the ground sample was placed in a small conical flask. Then 10 ml of 1% concentrated HCL in methanol was added. The flask was capped and continuously shaken for 20 min and the content was further centrifuged at 2500 rpm for 5 min. About 1.0 ml of the supernatant was pippetted into a test tube containing 5 ml of vanillin-HCL reagent.

Absorbance at 450 nm was read on spectrophotometer after 20 min of incubation at 30°C. A standard curve was prepared expressing the result as catechin equivalent as follows: Tannin (%) = C × 10 × 100/200. Where: C = Concentration corresponding to the optical density; 10 = volume of the extract (ml); 200= Sample weight (mg).

### Saponin determination

Five grams of plant sample was dispersed in 50 ml of 20% v/v ethanol prepared in distilled water. The suspension was heated over hot water bath for 4 h with continuous stirring at 55°C. The mixture was filtered and the residue re-extracted with another 50 ml of 20% ethanol. The combined extracts were reduced to 20 ml over hot water bath at about 9°C. The concentrated solution obtained was shaken vigorously with 10 ml of diethyl ether in a 250ml separating funnel; the aqueous layer was collected while the ether layer was discarded. The purification process and repeated. Twenty millilitre of but-1-ol was added to the filtrate and then washed twice with 10 ml of 5% w/v aqueous sodium chloride. The whole mixture was heated to evaporation on hot water bath and later oven dried at 40°C to a constant weight. The percentage saponins content of the sample was calculated using the formula described by Okwu and Josiah [27].

(1) $$\;\%\;\text{Saponins}=\frac{\text{Weight of final filtrate}}{\text{Weight of sample}}\times \;100$$

### Alkaloids determination

Alkaloids content of the plant sample was determined using the method described by Onyilagha and Islam [28]. Five gram of the powdered sample was weighed into a 250 ml beaker and 200 ml of 20% acetic acid in ethanol was added and covered to stand for 4 h. This was filtered and the extract was concentrated using a water bath to one-quarter of the original volume. Concentrated ammonium hydroxide was added drop wise to the extract until the precipitation was completed. The whole solution was allowed to settle and the collected precipitates were washed with dilute ammonium hydroxide and then filtered. The residue was dried and weighed. The alkaloid content was determined using this formula; $\%\;\text{Alkaloid}=\frac{\text{final weight of sample}}{\text{Initial weight of extract}}\text{×}\;100$.

### Ferric-reducing power (FRAP) assay

The reducing power of the extract was evaluated according to the method of Hemalatha and Kumar [29]. The mixture containing 2.5 ml of 0.2 M phosphate buffer (pH 6.6) and 2.5 ml of potassium ferricyanide (K3Fe(CN)6 (1% w/v) was added to 1mL of each of the extracts at different concentrations ranging from 0.025 – 0.5 mg/ml. The resulting mixtures were incubated at 50°C for 30 min, followed by the addition of 2.5 ml of trichloroacetic acid (10% w/v). The mixture was centrifuged at 3000 rpm for 10 min to collect the upper layer of the solution. A volume of 2.5 ml supernatant solution was mixed with distilled water (2.5 ml) and 0.5 ml of FeCl_3_ (0.1%, w/v). The absorbance was then measured at 700 nm against blank sample. Ascorbic acid and butylated hydroxyl toluene solution were used as positive controls. Increased absorbance of the reaction mixture indicated higher reducing power of the plant extract.

### Scavenging activity of 2, 2-Diphenyl-1-Picrylhydrazyl (DPPH) radical

The effect of extracts on DPPH radical was estimated using the method of Liyana-Pathiranan et al. [30]. About 0.1 ml of DPPH-methanol solution (0.135 mM) was mixed with 1.0 ml of different concentrations (0.025–0.5 mg/ml) of various extracts of *C. edulis*. The reaction mixture was vortexed thoroughly and left in the dark at room temperature for 30 min. The absorbance of the mixture was measured spectrophotometrically at 517 nm. Rutin and Butylated hydroxyl toluene (BHT) were used as standard drugs. The percentage of free radical scavenging was calculated according to the following equation: *%* scavenging = 100–(Abs sample–Abs blank)/Abs Control × 100.

### 2, 2'-azino-bis (3-ethylbenzthiazoline-6-sulphonic acid (ABTS) scavenging activity

The method of Re et al. [31] was adopted for the determination of ABTS activity of the plant extract. First the working solution was prepared by mixing two stock solutions of 7 mM ABTS solution and 2.4 mM potassium persulphate solution in equal amount and allowed to react for 12 h at room temperature in the dark. The solution was then diluted by mixing 1ml ABTS solution to obtain an absorbance of 0.706 ± 0.001 units at 734 nm using the spectrophotometer. Fresh ABTS solution was prepared for each assay. Plant extracts at different concentrations ranging from 0.025–0.5 mg/ml were allowed to react with 1 ml of the ABTS solution and the absorbance was taken at 734 nm after 7 min using the spectrophotometer. The ABTS scavenging capacity of the extract was compared with that of BHT and rutin. The percentage inhibition was calculated as ABTS scavenging activity of the extract using the following equation:

(2) $$\text{ABTS radical scavenging activity}={\text{Abs}}_{\text{control}}-{\text{Abs}}_{\text{sample}/}{\text{Abs}}_{\text{control}}\text{×}\;100$$

Where

Abs_control_ is the absorbance of ABTS radical+methanol;

Abs_sample_ is the absorbance of ABTS radical+sample extract/standard**.**

### Nitric oxide radical scavenging activity

The scavenging radical of nitric oxide was based on the procedure reported by Ebrahimzadeh et al. [11]. A volume of 2 ml of 10 mM sodium nitroprusside dissolved in 0.5 ml phosphate buffer saline (pH 7.4) was mixed with 0.5 ml of each plant extract or BHT or rutin at various concentrations (0.025-0.5 mg/ml). The mixture was incubated at 25°C for 150 min. An aliquot of 0.5 ml of the solution was withdrawn and mixed with 0.5 mL of Griess reagents [(1.0 ml sulfanilic acid reagent (0.33% in 20% glacial acetic acid at room temperature for 5 min with 1 ml of naphthylethylenediamine dichloride (0.1% w/v)]. The reaction mixture was incubated at room temperature for 30 min, after which absorbance was measured at 540 nm. The amount of nitric oxide radical was calculated using the equation: % inhibition of NO= A_0_−A_1/_A_0_ × 100, where A_0_ is the absorbance before reaction and A_1_ is the absorbance after reaction has taken place.

### Hydrogen peroxide scavenging activity

The ability of the plant extract to scavenge hydrogen peroxide was determined according to the method given by Karunakaran and Kumaran [24]. A solution of 4 mM H_**2**_O_**2**_ was prepared in phosphate buffer (0.1 M pH 7.4). Plant extract (4 ml) prepared in methanol at various concentrations (0.025–0.5 mg/ml) were mixed with 0.6 ml of 4 mM H_**2**_O_**2**_ solution prepared in phosphate buffer. The absorbance of hydrogen peroxide at 230 nm was determined after 10 min against a blank solution containing the plant extract without H_**2**_O_**2**_. The result obtained was compared with standard ascorbic acid. Percentage inhibition of H_**2**_O_**2**_ = Abs(control) – Abs(sample)/Abs(control) × 100, where, Abs (control): Absorbance of the control and Abs (sample): Absorbance of the extracts/standard.

### Statistical analysis

The experimental results were expressed as mean ± standard deviation (SD) of three replicates. Where applicable, the data were subjected to one way analysis of variance (ANOVA) to determine the significance difference in the extract used.

## Results and discussion

### Phytochemical screening

Qualitiative phytochemical analysis of the *C. edulis* leaf extracts revealed the presence of secondary metabolites in aqueous, ethanol, acetone and hexane extracts (Table 1).

**Table 1:** **Results of phytochemical screening of the extracts from *C. edulis *leaf**

**Phytochemicals**	**Aqueous**	**Ethanol**	**Acetone**	**Hexane**
Phenolics	+++	+++	+++	++
Flavonoids	+	+	+	+
Flavonols	+	+	+	+
Proanthocyanidins	+++	+++	+++	+++
Tannins	+++	+++	+++	++
Saponins	++	++	++	+
Alkaloids	++	++	++	+

Highly present-+++, Moderately present -++.

### Quantification of polyphenolic compounds

Due to the vast differences in the nature of the phytochemical constituents found in a plant, there is no particular solvent that is known to extract all the compounds on its own from the plant [23]. Therefore, in this study, we considered using hexane, acetone, ethanol and aqueous solvents for extraction to accommodate the range of polarities of the compounds present in *C. edulis* leaves. Our results showed that the choice of these various solvents played a crucial role in the quantitative analysis of different polyphenols extracted from the plant samples. The yield of different solvent extracts of *C. edulis* leaf is presented in Table 1.

From Table 1, we analysed all the values using a one way ANOVA test to verify if the phytochemical content in the four solvent extracts were significantly difference from each other at 95% confidence interval. From the overall ANOVA analysis, Table 2 [column Sig], there were significant differences amongst the solvent extracts. Therefore we proceeded in carrying out a multiple comparison test (LSD) using the “post-hoc” to analysed exactly where the differences among the extracts occur.

**Table 2:** **Quantitative analysis of the phytochemical evaluated from the leaf of *C. edulis***

	**Amount of phytochemical compounds in (mg/g)**			
	**Aqueous**	**Ethanol**	**Acetone**	**Hexane**
Phenols (TE/g)	517.71 ± 0.40 ^a٭^	330.87 ± 0.04 ^b^	557.11 ± 0.23 ^a^	64.14 ± 0.15 ^b^
Flavonoids (QE/g)	0.29 ± 0.01 ^b^	0.28 ± 0.01 ^b^	0.65 ± 0.04 ^b^	1.19 ± 0.041 ^b^
Flavonols (QE/g)	0.05 ± 0.001 ^a^	0.05 ± 0.001 ^a^	0.23 ± 0.05 ^a^	0.19 ± 0.03 ^a^
Proanthocy-anidins(CE/g)	896.7 ± 0.05 ^b^	115.28± 0.007 ^a^	753.87 ± 0.02 ^b^	134.91 ± 0.01 ^a^
Tannins (ND)	461 ± 0.07 ^a^	489 ± 0.28 ^b^	384 ± 0.14 ^a^	64 ± 0.14 ^b^
Saponins (ND)	34 ± 0.21 ^a^	45 ± 0.26 ^a^	11 ± 0.071 ^b^	2 ± 0.035 ^b^
Alkaloides (ND)	45 ± 0.06 ^b^	38 ± 0.02 ^b^	31 ± 0.021 ^b^	3 ± 0.014 ^b^

Values are expressed in mean ± standard deviation of the mean (n = 3). ND: not detected; (TE) tannic acid equivalent; (QE) quercetin equivalent; (CE) catechin equivalent.٭Values within a column followed by the same superscript are not significantly different at 95% confidence interval.

Quantification of compounds obtained from the crude extract varied greatly among the four solvents, which is an indication that solvents have different extracting capacity for polyphenols. As shown in Table 1, the concentration of phenol in the four solvent extracts is in the following decreasing order: acetone < aqueous < ethanol < hexane. The observed high phenol content in acetone (557.11 ± 0.228 mg/g) and aqueous (517.71 ± 0.015 mg/g) fractions is in agreement with what has been reported by other researchers [16]. However, there was no significant difference between acetone and water extracts at 95% confidence interval.

The value of flavonoid content in all the four solvent extracts was significantly different from each other at 95% confidence interval, Table 1. The solvent capacity follows the decreasing order of the extract hexane < acetone < aqueous < ethanol. Our results were contrary to the report of Syeda et al. [32] who reported that solvent extraction of total flavonoids followed a decreasing order of ethanol < methanol < acetone < ethyl acetate < dichloromethane < hexane.

In this study, the highest value of flavonols (0.23 ± 0.050 mg/g) was obtained from acetone extract, followed by hexane extract (0.19 ± 0.030 mg/g). The variation in the extraction capacity of the solvents could be due to the polarity of the solvent used, even though the differences were not significant.

An appreciable amount of proanthocyanidin was observed respectively in both aqueous (896.7 ± 0.005 mg/g) and acetone (753.89 ± 0.017 mg/g) extracts, while a moderate concentration was formed in hexane (134.91 ± 0.014 mg/g) and ethanol (115.28 ± 0.007 mg/g) extract, but the differences was not significant. Our results suggested that acetone and water are good solvent for extraction of bioactive compounds from plants as they gave the highest yield compared with other solvent used in this investigation.

Among the four solvent extracts for total tannins content, ethanol offered the best result (48.9 ± 0.283%), followed by aqueous extract (46.1 ± 0.071%), acetone extract (38.4 ± 0.141) and hexane (6.4 ± 0.141). Similar trends were found by Hanen et al. [19] for the total tannin contents using methanol extract, though the results obtained in this study was higher.

The percentage concentration of saponins in the ethanol extract (4.5 ± 0.262%) was not significantly different from the aqueous extract (3.4 ± 0.21%). Alkaloid content of the various extracts followed the trend: water < ethanol < acetone < hexane extracts [Table 1]. The results showed that aqueous extract exhibited the highest concentration of alkaloids compared to other solvents.

Nevertheless, our present results on *C. edulis* phytochemicals showed parallel trends in the behaviour of same family species revealed by Hanen et al. [19].

### Reducing power

Generally, polyphenol are known to be major plant compounds and they have been reported to have multiple biological effects, including antioxidant activity. Their antioxidant activity is mainly due to their redox properties, hydrogen donors and singlet oxygen quenchers, which can play an important role in adsorbing and neutralizing free radicals [33,34]. The importance of the antioxidant constituents of plant materials in the maintenance of health and protection from heart disease or cancer is also raising interest among scientists, food manufacturers, and consumers [35].

**Figure 1:**
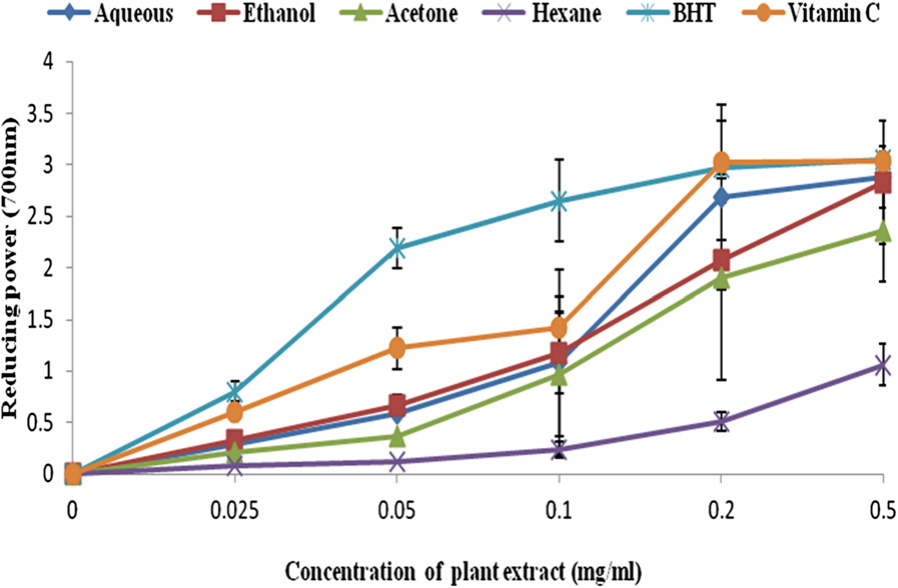
**Reducing power of the various extract of *C. edulis *in comparison to BHT and ascorbic acid. **n = 3. Error bars indicate standard deviation.

The antioxidant activity of the four plant extract were investigated by measuring the transformation of Fe^3+^/ferricyanide complex to Fe^2+^/ferrous form [36]. In this study we observed a concentration-dependent increase in the absorbance of reaction mixture for all the solvent extracts and the standard drugs (BHT and Ascorbic acid) [Figure 1. At 0.2 – 0.5 mg/ml, aqueous extract exhibited the highest reducing power that ranges from (2.69 ± 0.13 – 2.88 ± 0.05), followed by ethanol (2.07 ± 0.02 – 2.83 ± 0.06), acetone (1.91 ± 0.28 – 2.36 ± 0.25) and hexane (0.51 ± 0.18 – 1.06 ± 0.018) when compared with BHT (2.97 ± 0.04 – 3.05 ± 0.09) and ascobic acid (3.03 ± 0.08 – 0.04 ± 0.09) respectively. The observed reducing ability of the plant extracts might be due to the presence of hydrophilic polyphenolic compounds [11,37] These results are in full agreement with the previous studies which reported that the reducing power of plant extracts correlated with the phenolic content [36-38]. The reducing capability increase in the following order: Ascorbic acid < BHT < aqueous < ethanol < acetone < hexane.

### Scavenging assay of the four solvent extracts against DPPH

**Figure 2:**
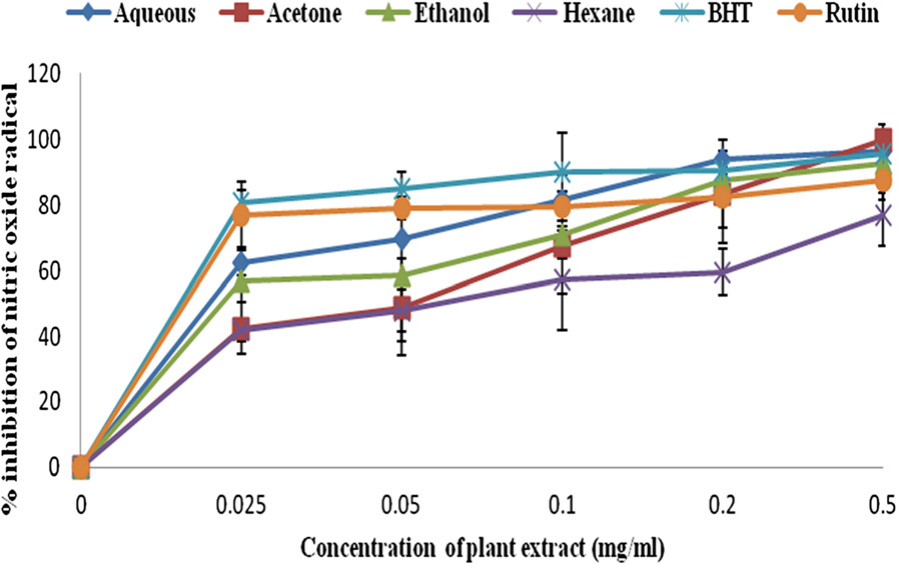
**DPPH radical scavenging activity of the various extracts of *C. edulis *in comparison to BHT and rutin drug. **n = 3. Error bars indicate standard deviation.

The free radicals of DPPH contains an odd electron, which is responsible for the deep purple colour [39]. When DPPH accept an electron donated by an antioxidant compound, it is decolorized which can be quantitatively measured from the changes in absorbance [40]. This was observed in our experiment immediately the colour changing from purple to yellow, indicating that the odd electron of DPPH radical is paired with hydrogen from a free radical scavenging antioxidant to form the reduced DPPH-H at 517 nm. Figure 2 illustrate the percentage inhibition of all the extracts in the following order: aqueous < ethanol < acetone < hexane.

At the lowest concentration of 0.025 mg/ml, aqueous extract was comparable with that of the standard drugs BHT (90 ± 0.014) with IC_50_ value of 0.015 mg/ml and rutin (91.4 ± 0.006) with IC_50_ value of 0.018 mg/ml. Thus these comparisons indicate that aqueous and ethanol extracts possess high DPPH scavenging activity at the lowest concentration (0.025 mg/ml). This is an indication that *C. edulis* can serve as a potential natural antioxidant over standard drugs.

### Scavenging assay of the four solvent extracts against ABTS radicals

**Figure 3:**
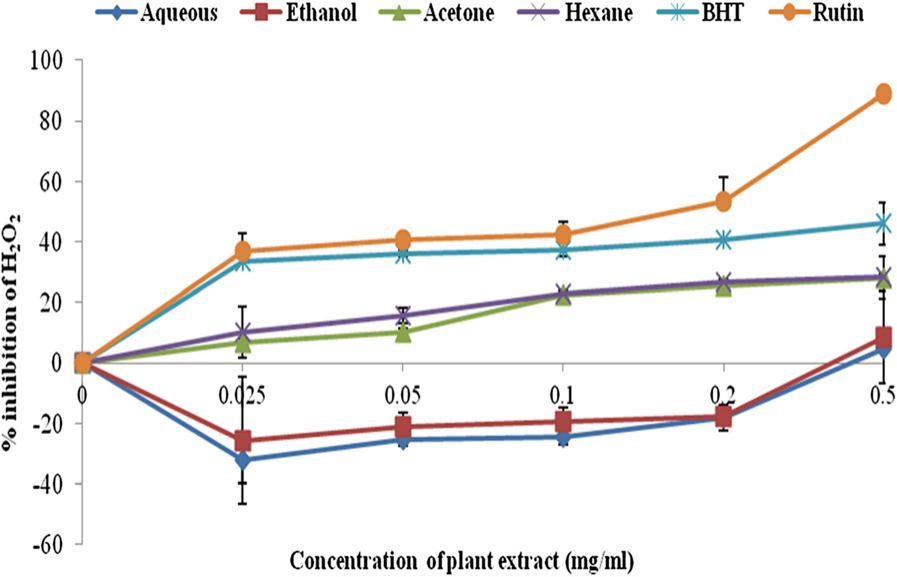
**ABTS radical scavenging activity of the various extracts of *C. edulis *in comparison to BHT and rutin drug. **n = 3. Error bars indicate standard deviation.

In this study, the percentage inhibition of ABTS radical scavenging activity was concentration-dependent with increased in the reaction mixture for all the extracts including the standard drugs [Figure 3. At the lowest concentration dose response of 0.025 mg/ml, only ethanol extract exhibited the highest percentage inhibition of 89.35 ± 7.07 with IC_50_ value of 0.023 mg/ml. This was found higher than the standard drugs BHT (80.6 ± 0.56, IC_50_ value of 0.023 mg/ml and rutin (77.0 ± 0.36, IC_50_ value of 0.024 mg/ml). The remaining three extracts showed varied levels of ABTS radical scavenging activity [Figure 3. The aqueous extract showed minimum absorbance of 39.62 ± 0.0066, IC_50_ value of 0.05 mg/ml followed by acetone (13.5 ± 0.0014, IC_50_ value of 0.05) and hexane (6.21 ± 0.0007, IC_50_ value of 0.1 mg/ml). This implies that at the lowest concentration dose-respond, this plant extract could serve as free radical inhibitors [18]. The finding obtained in this study is similar to the recent study by Olubunmi and Afolayan [16] who reported that compounds with higher amounts of polar solvents possesses the ability to inhibit both DPPH and ABTS radicals as compared with non polar solvents [18].

### *C. edulis* extracts against nitric oxide activity

**Figure 4:**
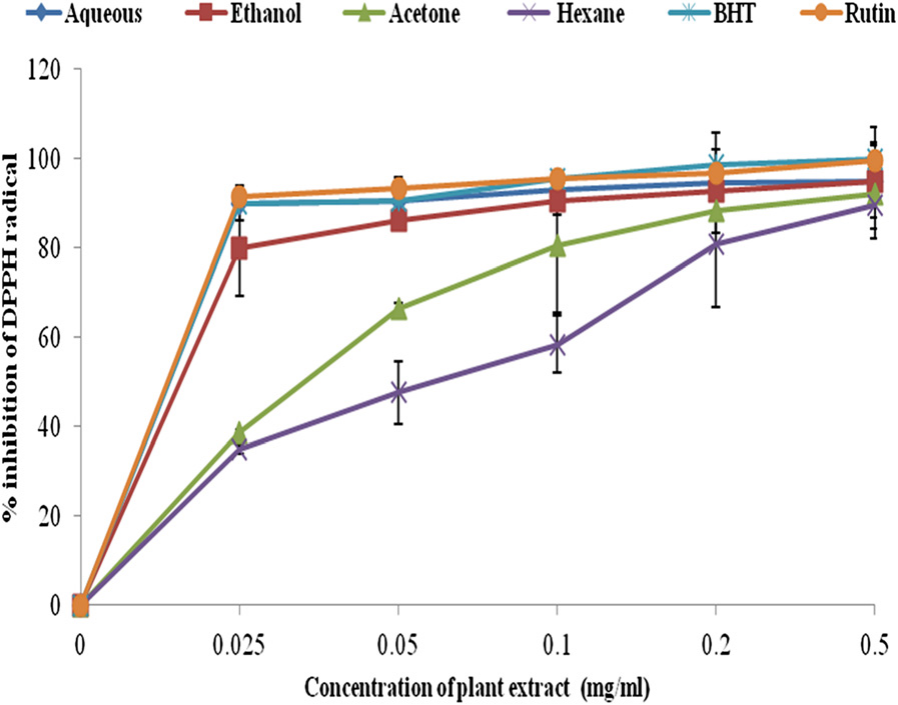
**Nitric oxide scavenging activity of the various extract of *C. edulis *in comparison to BHT and rutin drugs. **n = 3. Error bars indicate standard deviation.

Eradication of nitric oxide by the four solvent extracts was shown in Figure 4. Out of the four extracts investigated, only aqueous and ethanol extracts showed the highest percentage nitric oxide inhibition of 62.34 ± 0.004 and 42.5 ± 0.001 at a very low concentration of 0.025 mg/ml repectively. The minimum inhibitory concentration required to reduce the nitric oxide radicals by 50% of aqueous extract was 0.018 mg/ml while that of ethanol extract was 0.016 mg/ml concentration. This was significantly similar to the concentration needed for commercial rutin drug (0.015 mg/ml) and BHT (0.012 mg/ml).

Acetone and hexane extracts had activity at 0.062 and 0.025 mg/ml respectively. Lower absorbance (IC_50_) of the reaction mixture indicates higher free radical scavenging activity [41]. Therefore, it can be stated that ethanol extract possess strong antioxidant activity. The nitric oxide scavenging activities of the four extracts decreases in this order: BHT < rutin < ethanol < aqueous < hexane < acetone extracts.

Inhibitory activity of nitric oxide by Olubunmi and Afolyan [16] and Banerjee et al. [42] reported that solvent with same polarity, such as ethanol and methanol always produced the best activity.

### Inhibitory activity of the four extracts against hydrogen peroxide

Hydrogen peroxide (H_2_O_2_) is a well-known antimicrobial agent with cleansing property when it was first introduced into clinical practice [42]. In recent times it has lost that favour as a result of its toxicity effect in the human cells [43]. As a liquid, H_2_O_2_ is usually used as alternative for conventional western medicine to treat patients. Occasionally, it can also be mixed with water to cure skin infection or dirty wounds [44].

**Figure 5:**
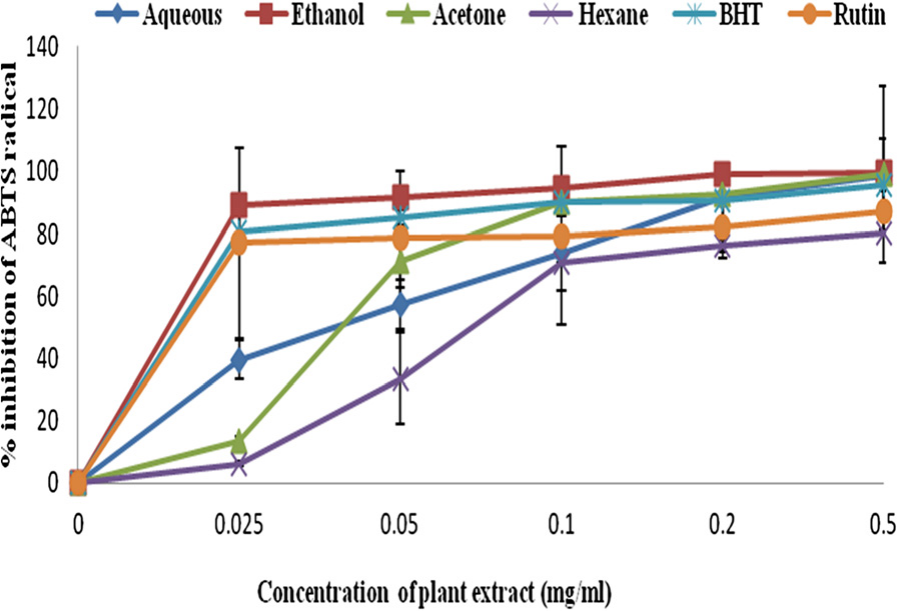
**Percentage inhibition of hydrogen peroxide scavenging activity of various extracts in comparison to BHT and rutin drugs. **n = 3. Error bars indicate standard deviation.

Figure 5 showed that the four solvent extracts of *C. edulis* demonstrated a strong scavenging activity against H_2_O_2_. At a very low concentration of 0.025 mg/ml we observed a concentration dependant decrease in H_2_O_2_ activity. A very weak inhibitory activity was found in both aqueous and ethanol extracts (4.6 ± 0.007 and 8.6 ± 0.015). The highest concentration was found at 0.5mg/ml [Figure 5. The best percentage scavenging activity was shown by hexane extract (28.3 ± 7.07) and the IC_50_ value was 0.3mg/ml, followed by acetone (28.2 ± 0.005) with IC_50_ value of 0.5 mg/ml. However, their activity was not significantly different at 95% confidence interval. BHT and rutin were used as standard drugs with percentage inhibition of 46.1 ± 70.07 and 88.9 ± 0.01, with IC_50_ of 0.025 and 0.029 mg/ml respectively. The ability of acetone and hexane extracts to scavenge H_2_O_2_ has been reported by Olubunmi and Afolayan [16] and Kirmizigual et al. [45].

## Conclusion

In summary, *C. edulis* extracts appear to possess compounds with antioxidant properties, thus justifying its traditional usage for the management of common infections in HIV/AIDS patients. Overall, both aqueous and ethanol were found to be the best solvents for antioxidant activity. We will conduct further research to isolate and identify the active compounds, and to determine their exact mechanism of action.

## Abbreviations

*C. edulis*: *Carpobrotus edulis*; DPPH: 1,1- Diphenyl-2-Picrylhydrazyl; ABTS: 2,2’-Azino-Bis(3-ethylbenzthiazoline-6-sulfonic acid); H_2_O_2_: Hydrogen Peroxide; NO: Nitric Oxide; ROS: Reactive Oxygen Species; OH-: Hydrogen Anion; O2-: Dioxygen; RO2-: Peroxyl Radical; LOO-: Lipid Peroxyl Radical; DNA: Deoxyribonucleic Acid; BHT: Butylhydroxytoluene; Na_2_CO_3_: Sodium Carbonate; AlCl_3_: Aluminium chloride; HCL: Hydrochloric Acid; K3Fe(CN)6: Potassium Ferricyanide; FeCl3: Potassium Chloride; AA: Antioxidant Activity; ANOVA: Analysis Of Variance; TE: Tannic acid Equivalent; QE: Quercetin Equivalent; CE: Catechin Equivalents; LSD: Least Significant Difference.

## Competing interests

The authors declare that they no competing interests.

## Authors contributions

BEO was responsible for the collection of plant materials from the traditional healers, carried out all experiments, performed data analysis and drafted the manuscript. GB edited the manuscript. AJA participated in study design, coordinated the plant material storage, supervised in the laboratory assay and made substantial contribution to revise the manuscript critically. All authors read and approved the final manuscript.

## Pre-publication history

The pre-publication history for this paper can be accessed here:

http://www.biomedcentral.com/1472-6882/12/215/prepub
